# Real-world safety of rapid 30-minute intravenous isatuximab infusion in multiple myeloma: results from the largest published cohort

**DOI:** 10.3389/fonc.2026.1906850

**Published:** 2026-07-09

**Authors:** Xabier Gutiérrez López De Ocáriz, Ernesto Pérez Persona, Laura Salcedo Cuesta, Eusebio Martín Chacón, Maialen Sirvent Auzmendi, Pamela Millacoy Austerrritt, Marcos Lorenzo Pérez, Elena Landete Hernández, Vicente Carrasco Baraja, Irene Sanchéz Romero, Tania Ivett Arámbula, Ianire Etxeguren Urkixo

**Affiliations:** 1 Servicio de Hematologia (Hematology Department), Araba University Hospital, Vitoria-Gasteiz, Spain; 2 Servicio de Hematologia (Hematology Department), Hospital Universitario de Navarra, Pamplona, Spain; 3 Servicio de Hematologia (Hematology Department), Hospital Universitario Virgen del Rocio, Seville, Spain; 4 Servicio de Hematologia (Hematology Department), Hospital Universitario de Donostia, Donostia-San Sebastian, Spain; 5 Servicio de Hematologia (Hematology Department), Hospital Alvaro Cunqueiro, Vigo, Spain; 6 Servicio de Hematologia (Hematology Department), Hospital Universitario Infanta Leonor, Madrid, Spain; 7 Servicio de Hematologia (Hematology Department), Hospital Royo Villanova, Zaragoza, Spain; 8 Servicio de Hematologia (Hematology Department), Hospital Universitario de Valme, Seville, Spain; 9 Servicio de Hematologia (Hematology Department), Hospital de Galdakao-Usansolo, Galdakao, Spain

**Keywords:** day hospital, infusion-related reaction, isatuximab, multiple myeloma, rapid infusion, real-world evidence

## Abstract

**Background:**

Isatuximab, an anti-CD38 monoclonal antibody, is a cornerstone therapy for multiple myeloma (MM). Standard intravenous infusion requires a minimum of 75 minutes per dose, representing a significant burden on patients and day-hospital resources. A rapid 30-minute infusion protocol has shown promise in early clinical trials, but large real-world datasets remain scarce.

**Methods:**

We conducted a descriptive, retrospective, multicenter study across 9 Spanish hospitals, including patients with confirmed MM who had received at least one rapid (30-minute) intravenous isatuximab infusion. Demographic, clinical, treatment, and safety data were collected. Infusion-related reactions (IRRs) were graded per CTCAE v5.0. Data cut-off: 1 May 2026.

**Results:**

A total of 103 patients were included (median age 66 years; 54.4% female). A total of 1,344 rapid 30-minute infusions were administered. Only 1 IRR was recorded: per-patient rate 0.97% (95% CI: 0.17%–5.30%), per-infusion rate 0.074% (95% CI: 0.013%–0.420%). Notably, 22 patients (21.4%) had experienced IRRs at standard rate; 21 of these (95.5%) completed the rapid protocol without reverting to standard rate. No patient permanently discontinued isatuximab due to an IRR. No treatment failure was attributable to the infusion rate change during the observation period. The protocol generated an estimated 1,008 hours of cumulative day-hospital chair time savings.

**Conclusion:**

Rapid 30-minute intravenous isatuximab demonstrates an excellent real-world safety profile, including in patients with prior IRRs, and generates clinically meaningful resource savings. To our knowledge, this is the largest published real-world cohort to date. These findings support its use as a feasible implementation strategy while prospective comparative studies remain warranted.

## 1 Introduction

Isatuximab, an anti-CD38 monoclonal antibody, is a cornerstone of treatment for relapsed/refractory multiple myeloma (MM) in combination with carfilzomib-dexamethasone (Isa-Kd) or pomalidomide-dexamethasone (Isa-Pd), and is now entering first-line regimens following recent regulatory approvals.

The standard intravenous administration requires a fixed volume of 250 mL infused at a maximum rate of 200 mL/h, resulting in a minimum infusion duration of 75 minutes per dose (1). Given the chronic and often lifelong nature of MM treatment, this represents a substantial burden on patients, caregivers, and day-hospital resources. A rapid 30-minute infusion protocol has been evaluated in early clinical trials with encouraging tolerability data (2), but large real-world datasets remain scarce.

Here, we present results from the largest published real-world cohort of patients receiving rapid 30-minute isatuximab infusions, from a multicenter retrospective study across 9 Spanish hospitals.

## 2 Methods

We conducted a descriptive, retrospective, multicenter study including patients with a confirmed MM diagnosis who had received at least one rapid (30-minute) intravenous isatuximab infusion at any of the 9 participating centers. Demographic, clinical, treatment, and safety data were collected. Infusion-related reactions (IRRs) were graded according to CTCAE v5.0. The study was approved by the relevant Ethics Committee (CEIm). The data cut-off date was 1 May 2026. Given the retrospective nature of the study, missing data were not imputed; available data were analyzed as recorded, and the potential for information bias and underreporting of mild IRRs is acknowledged as a limitation. The rapid infusion protocol followed that described by Ocio et al. (HemaSphere 2024;8:e70041). Isatuximab 10 mg/kg was diluted in a fixed volume of 250 mL of 0.9% sodium chloride. The first rapid infusion was administered at a rate of 250 mL/h; in the absence of infusion-related reactions, subsequent infusions were administered at a fixed rate of 500 mL/h over 30 minutes. Patients were considered eligible for rapid infusion after tolerating at least one prior standard-rate infusion without significant IRR, in accordance with the criteria of Ocio et al.; in practice, the majority of patients had completed at least one full standard cycle (i.e., ≥4 infusions) before switching, with a median of 6 prior standard-rate infusions (range 1–59) across the cohort. All patients received standard premedication prior to each infusion as per the isatuximab Summary of Product Characteristics (SmPC), consisting of antihistamines, antipyretics, and corticosteroids; no systematic intensification of premedication was applied. Patients were observed for 20–30 minutes following the first two rapid infusions. The same protocol was applied uniformly across all 9 participating centers.

## 3 Results

### 3.1 Patient characteristics

A total of 103 patients were included. The median age was 66 years (range 45–83); 54.4% were female. High-risk cytogenetic features were present in 41.7% of patients, and 30.1% had received a prior anti-CD38 antibody. The most common regimen was Isa-Kd (68.0%), followed by Isa-VRd (15.5%) and Isa-Pd (13.6%). Full patient characteristics are presented in **Table 1**.

**Table 1:** Patient characteristics and infusion safety data (N = 103).

Variable	N = 103
Demographics	
Age, median (range), years	66 (45–83)
Female sex, n (%)	56 (54.4%)
Renal function (eGFR), median (range), mL/min	86 (6–111)
Disease staging & performance status	
ISS stage I, n (%)	39 (37.9%)
ISS stage II, n (%)	12 (11.7%)
ISS stage III, n (%)	27 (26.2%)
ISS not available, n (%)	25 (24.3%)
ECOG PS 0–1, n (%)	81 (78.6%)
Cytogenetic risk	
High-risk cytogenetics, n (%)	43 (41.7%)
Standard-risk cytogenetics, n (%)	54 (52.4%)
Not available, n (%)	6 (5.8%)
Prior treatment	
Number of prior lines, median (range)	1 (0–5)
Treatment-naïve (1st line), n (%)	13 (12.6%)
1 prior line, n (%)	56 (54.4%)
Prior anti-CD38 antibody, n (%)	31 (30.1%)
Isatuximab-based regimen	
Isa-Kd, n (%)	70 (68.0%)
Isa-VRd, n (%)	16 (15.5%)
Isa-Pd, n (%)	14 (13.6%)
Standard-rate infusions (prior to transition)	
Median prior standard-rate infusions (range)	10 (1–59)
IRR at standard rate, n (%)	22 (21.4%)
Grade 1, n (%)	11 (50.0%)
Grade 2, n (%)	11 (50.0%)
Rapid iInfusion (30-min)	
Total rapid infusions, n	1,344
Median rapid infusions per patient (range)	10 (2–41)
IRR during rapid infusion, n (%)	1 (1.0%)
Permanent discontinuation due to IRR, n (%)	0 (0%)

IRR, infusion-related reaction; ISS, International Staging System; ECOG PS, Eastern Cooperative Oncology Group performance status; eGFR, estimated glomerular filtration rate; Isa-Kd, isatuximab-carfilzomib-dexamethasone; Isa-Pd, isatuximab-pomalidomide-dexamethasone; Isa-VRd, isatuximab-bortezomib-lenalidomide-dexamethasone.

### 3.2 Standard-rate infusion history

Prior to protocol transition, patients had received a median of 6 standard-rate infusions (range 1–59). Of note, 22 patients (21.4%) had experienced an IRR at standard rate (grade 1: 50.0%; grade 2: 50.0%). Of these 22 patients, 21 (95.5%) successfully completed the rapid infusion protocol without reverting to standard rate. The single IRR recorded during rapid infusion occurred in one patient from this subgroup — a patient who had experienced a grade 1 IRR during the first standard-rate isatuximab infusion only, with all subsequent standard-rate infusions tolerated without incident. No systematic intensification of premedication was applied; standard SmPC premedication was used throughout. No center-specific differences in the management of patients with prior IRR were identified, although the retrospective design limits the ability to formally exclude such variability.

### 3.3 Safety of rapid 30-minute infusion

A total of 1,344 rapid 30-minute infusions were administered, with a median of 10 per patient (range 2–41). Only 1 IRR was recorded: a per-patient rate of 0.97% (95% CI: 0.17%–5.30%) and a per-infusion rate of 0.074% (95% CI: 0.013%–0.420%), calculated using the Wilson score method. The reaction was grade 1 in severity and managed by adjusting premedication while maintaining the rapid infusion rate, with no need to interrupt or revert to standard rate. No patient permanently discontinued isatuximab due to an IRR.

### 3.4 Efficacy and resource utilization

No treatment failure was considered attributable to the change in infusion duration during the available observation period. Formal efficacy endpoints were not assessed before and after the transition, and this study was not designed to evaluate anti-myeloma efficacy.

From a resource utilization standpoint, the 45-minute saving per infusion resulted in an estimated cumulative saving of over 1,008 hours of day-hospital chair time across the entire cohort (1,344 infusions × 45 min). This represents an arithmetic estimate based on infusion duration alone and does not constitute a formal health-economic analysis. Meaningful scheduling gains and earlier patient discharge were noted. Patient and caregiver satisfaction was reported informally by treating physicians during routine clinical follow-up; no formal satisfaction assessment instrument was applied.

## 4 Discussion

Our results confirm the excellent safety and feasibility of the rapid 30-minute isatuximab infusion protocol in a large, heterogeneous real-world population. To our knowledge, this is the largest published cohort to date, substantially exceeding the recent Canadian single-center series of 15 patients (127 infusions) reported by Kotb et al. (3).

A notable observation is the difference in IRR rate between the two phases: 0.97% per patient (0.074% per infusion) with the rapid protocol versus 21.4% during standard-rate infusion. This comparison must be interpreted with caution and should not be construed as a causal reduction attributable to the rapid protocol. Patients had received a median of 6 prior standard-rate infusions before switching, and IRRs are well-known to be more frequent during early administrations. This is therefore not a head-to-head comparison, and the difference is subject to both selection bias and timing bias. We present this as a descriptive observation. Nevertheless, the very low IRR rate during rapid infusion, including in patients with prior IRRs at standard rate, is clinically reassuring.

Importantly, 21 of the 22 patients (95.5%) with prior IRR to standard-rate infusions successfully completed the rapid protocol without reverting to standard rate, supporting its feasibility even in this potentially higher-risk subgroup. The multicenter design across 9 geographically diverse institutions strengthens the generalizability of these findings. With subcutaneous and on-body injector isatuximab strategies emerging, rapid intravenous infusion should be positioned as a feasible, low-complexity, cost- and capacity-conscious implementation strategy, particularly relevant during the transition phase before subcutaneous formulations achieve broad availability, reimbursement, and workflow integration across healthcare systems.

The resource impact deserves emphasis. The 1,008 hours of cumulative chair time saved represents a substantial organizational gain — equivalent to over 42 full days of day-hospital capacity. As isatuximab-based regimens expand into first-line treatment of both transplant-eligible and transplant-ineligible patients (4, 5), this figure will increase considerably. Beyond logistics, the reduction in infusion time has a direct positive impact on quality of life for patients and caregivers.

This study has inherent limitations, including its retrospective design and lack of a comparator arm. Formal efficacy endpoints were not measured before and after the protocol switch. The absence of systematic follow-up duration data from the start of rapid infusion and the lack of a prospective protocol for managing patients with prior IRR limit the conclusions that can be drawn. Missing data were not imputed, and the potential for information bias and underreporting of mild events is acknowledged. Nevertheless, no treatment failure attributable to the infusion rate modification was observed during the available observation period.

In conclusion, rapid 30-minute intravenous isatuximab demonstrates an excellent safety profile in real-world practice, including in patients with prior IRR, and generates clinically meaningful resource savings. These findings support its use as a feasible, pragmatic implementation strategy in centers where subcutaneous formulations are not yet available or reimbursed, while prospective comparative studies remain warranted.

## Data Availability

The original contributions presented in the study are included in the article/supplementary material. Further inquiries can be directed to the corresponding author.
